# A Look into Stem Cell Therapy: Exploring the Options for Treatment of Ischemic Stroke

**DOI:** 10.1155/2017/3267352

**Published:** 2017-10-22

**Authors:** Cesar Reis, Michael Wilkinson, Haley Reis, Onat Akyol, Vadim Gospodarev, Camila Araujo, Sheng Chen, John H. Zhang

**Affiliations:** ^1^Department of Physiology and Pharmacology, Loma Linda University School of Medicine, 11041 Campus Street, Risley Hall, Room 219, Loma Linda, CA 92354, USA; ^2^Loma Linda University School of Medicine, Loma Linda, CA 92354, USA; ^3^Department of Neurosurgery, Second Affiliated Hospital, School of Medicine, Zhejiang University, Hangzhou, China; ^4^Department of Neurosurgery, Loma Linda University School of Medicine, Loma Linda, CA 92354, USA

## Abstract

Neural stem cells (NSCs) offer a potential therapeutic benefit in the recovery from ischemic stroke. Understanding the role of endogenous neural stem and progenitor cells under normal physiological conditions aids in analyzing their effects after ischemic injury, including their impact on functional recovery and neurogenesis at the site of injury. Recent animal studies have utilized unique subsets of exogenous and endogenous stem cells as well as preconditioning with pharmacologic agents to better understand the best situation for stem cell proliferation, migration, and differentiation. These stem cell therapies provide a promising effect on stimulation of endogenous neurogenesis, neuroprotection, anti-inflammatory effects, and improved cell survival rates. Clinical trials performed using various stem cell types show promising results to their safety and effectiveness on reducing the effects of ischemic stroke in humans. Another important aspect of stem cell therapy discussed in this review is tracking endogenous and exogenous NSCs with magnetic resonance imaging. This review explores the pathophysiology of NSCs on ischemic stroke, stem cell therapy studies and their effects on neurogenesis, the most recent clinical trials, and techniques to track and monitor the progress of endogenous and exogenous stem cells.

## 1. Introduction

Ischemic stroke accounts for 87% of all stroke events and is the 5th leading cause of death in the United States. The National Stroke Association estimates that there are nearly 7 million stroke survivors and though functional mobility impairments exist on a spectrum, it is a leading cause of adult disability [1]. It is well understood that stem cells are the building blocks of life. Achieving guidance of stem cells towards regenerating neurons and damaged tissue caused by ischemic stroke is a new and innovative area of research currently being investigated [2]. Endogenous neural stem and progenitor cells (NSPCs), also described in this review as neural stem cells (NSCs), persist in the subventricular zone (SVZ) lining the ventricles and the subgranular zone (SGZ) of the hippocampus in the adult brain. Finding ways to mobilize and induce neurogenesis in an area of focal ischemia is an area of current research [3]. Though not yet FDA approved for treatment of acute and chronic stroke, clinical trials are well underway to demonstrate their therapeutic benefits.

Various methods of stem cell therapy are being explored using animal models including the use of endogenous and exogenous stem cells. Interestingly, exogenous stem cells have been shown to induce endogenous NSCs towards neuronal differentiation [4, 5]. Cotransplantation therapy is another aspect of stem cell research that offers promising effects on neuronal differentiation and survival. One study looked at transplanting astrocytes with NSCs and found a higher ratio of survival and proliferation compared with transplanting NSCs alone [6]. Embryonic stem cells show positive therapeutic effects in animal models, as studies have determined that they can focus on regions that support neural differentiation within the adult brain, such as the substantia nigra pars compacta. [7] This aspect of stem cell therapy has unique benefits worth translating into the clinical setting.

Lastly, finding a tracking method to follow the stem cells on their path to neurogenesis provides clinicians with knowledge on the progress of the stem cells, including where they are mobilizing and proliferating [8]. In light of the vast amount of animal model research conducted in recent years, progressing to clinical trials has shown to be challenging, yet promising. The Pilot Investigation of Stem Cells and Stroke (PISCES) clinical trial injected a NSC drug into the ipsilateral putamen following ischemic insult and recorded images and clinical progress over a two-year span. The study found improvement in neurological function and no major adverse events [9]. Uncovering the intricacies and challenges of stem cell therapy using animal models for a variety of stem cell types prepares the medical community for more clinical trials like PISCES and future use of stem cells as a primary treatment option for patients recovering from ischemic stroke.

## 2. Pathophysiology of Ischemic Stroke

Stroke is caused by a critical disruption of blood supply in a specific area of the brain, resulting from either a sudden or slowly progressing obstruction of a major brain vessel, often leading to death or permanent neurological deficits [10]. Hemorrhagic stroke is caused by rupture of blood vessels in the brain, while ischemic stroke from embolism, thrombolysis, or cryptogenic mechanisms interrupts blood supply to the brain and is responsible for the vast majority of strokes seen in patients (87%) [11]. A lack of blood supply to the ischemic area of the brain known as the penumbra initiates an ischemic cascade whereby brain function stops if oxygen deprivation exceeds 60 to 90 seconds and brain tissue dies within 3 hours of anoxia leading to cerebral infarction. It is within the penumbra that many therapeutic interventions are targeted since its salvage is directly related to recovery [12]. Of the different types of cells found within the brain, neuronal cells are the most vulnerable to changes in oxygen content and can quickly become dysfunctional and die [13]. In an attempt to maintain cellular energy levels, neurons resort to anaerobic metabolism producing substantially less energy in the form of adenosine triphosphate than they would with normal aerobic glycolytic mechanisms. In addition, toxic byproducts including lactic acid are released, further disrupting the acid/base balance leading to additional cellular stress and death [14].

The highly coordinated cellular consequences after ischemic stroke include excitotoxicity, mitochondrial dysfunction, and oxidative stress due to a large intracellular influx of Ca^2+^ ions following disruption of transmembrane protein channels. Ischemia-induced reductions in nutrient availability for neuronal cells lead to the overproduction of excitatory amino acids, namely, glutamate, due to a disruption in the ionic gradients. N-Methyl-D-aspartate (NMDA) glutamate receptors induce increased amounts of intracellular Ca^2+^ influx leading to activation of Ca^2+^-dependent enzymes including proteases, calpain, and caspases, thereby setting off mitochondrial mechanisms of apoptosis and necrosis [15]. Neural circuitry is subsequently disrupted due to chronic stimulation of glutamate that can persist for months. This overabundant Ca^2+^ influx leads to activation of caspase-dependent cellular death pathways involving caspase-12, caspase-9, and caspase-3 due to the release of cytochrome C from mitochondria. Furthermore, important reactive oxygen species upregulated by Ca^2+^ influx in the mitochondria are implicated in reperfusion injury after ischemia leading to necrosis [16]. Free radicals, including the NO byproduct peroxynitrite, leads to oxidative damage through inhibition of signal transduction cascades favoring cell death mechanisms and inhibiting recovery from ischemic injury [17]. Dying neural cells release signals activating proinflammatory pathways leading to post ischemic inflammation that plays a role in activating the immune response.

## 3. Understanding Endogenous NSCs

Within the last decade, neurogenesis from endogenous NSCs has shown potential in ameliorating ischemic brain tissue following ischemic stroke through regenerative efforts. The fate of endogenous NSPC is quite complex and depends on many factors but takes on four general processes including proliferation, migration, cell survival, and neuronal differentiation [18]. Here, we discuss the process of how endogenous NSPCs provide neural progenitors for hippocampal and olfactory neurogenesis under normal physiological conditions.

### 3.1. Proliferation

**Figure 1:**
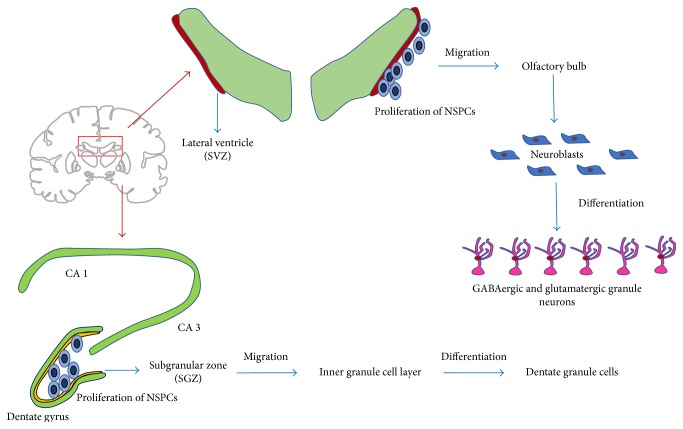
This figure demonstrates neurogenesis of endogenous neural stem cells. (1) Neurogenesis and proliferation occur in the SVZ and SGZ on the lateral ventricle and hippocampus, respectively. (2) NSPC migration occurs through the rostral migratory stream to the olfactory bulb, where neuroblasts migrate as interneurons through specific cell layers. From the SGZ, NSPCs migrate to the inner granule cell layer. (3) Differentiation occurs once neuroblasts reach glomeruli within the olfactory bulb or the inner granule cell layer. The majority of SVZ-derived neuroblasts become GABAergic granule neurons. After complete differentiation and maturation of neuroblasts from the SGZ, new neurons possess GABAergic and glutamatergic characteristics.

Neurogenic regions in the adult brain have been localized in two areas, the SVZ of the lateral ventricles and SGZ in the dentate gyrus of the hippocampus [19, 20]. It has been shown that the specific microenvironment in which the progenitor cell is located plays a major role in neurogenesis, as those residing in the SVZ and the SGZ are the only cells capable of becoming neurons without the use of extrinsic factors to assist (Figure 1). In addition, should these cells be relocated to another region of the brain, they differentiate into oligodendrocytes and astrocytes, further supporting the idea that NSPCs outside the SVZ and SGZ will most likely undergo glial rather than neuronal differentiation [21].

### 3.2. Migration

Understanding the migration pathway of NSCs allows for comparison of the migration process that happens under ischemic conditions. In the adult SVZ, radial glia-like cells lead to the production of transient amplifying cells which produce neuroblasts that will form a chain and migrate through the rostral migratory stream (RMS) towards the olfactory bulb within an astrocyte-derived tube [22]. Stromal cell-derived factor-1 (SDF-1), also known as an angiogenetic cytokine, has been reported to function in conducting neuroblast migration within the RMS. Chemokine-induced NSPC migration necessitates extracellular matrix remodeling via activation of matrix metalloproteinases that are functionally active along the SVZ-olfactory bulb pathway [23]. The main olfactory bulb includes principal neurons as well as local circuit neurons, and the location where olfactory axon terminals contact the principal and local circuit neurons is called the glomeruli [24]. In the olfactory bulb, the neuroblasts migrate as interneurons through specific cell layers towards glomeruli where differentiation ultimately occurs [25]. In the adult SGZ, both radial and nonradial precursors generate neuroblasts that then migrate to the inner granule cell layer of the hippocampus where they have been shown to differentiate into dentate granule cells (Figure 1) [26].

### 3.3. Survival and Differentiation

Recent studies suggest that the SVZ is highly organized, with each area having specific stem cells with unique neuronal fates. In the olfactory bulb, the majority of SVZ-derived neuroblasts become axon-less GABAergic granule neurons while a minority become GABAergic periglomerular neurons and even fewer become short-axon glutamatergic juxtaglomerular neurons [25]. Once arriving in the inner granule cell layer of the hippocampus from the SGZ, new dentate granule cells are generated. Local interneurons tonically release GABA, activating dendritic formation and extensions into the molecular layer. GABAergic synaptic inputs and glutamatergic synaptic inputs further develop. After complete maturation, the new neurons possess similar firing behavior, amplitude, and kinetics of both GABAergic and glutamatergic inputs (Figure 1) [27].

## 4. Neurogenesis following Ischemia: Endogenous NSCs

**Figure 2:**
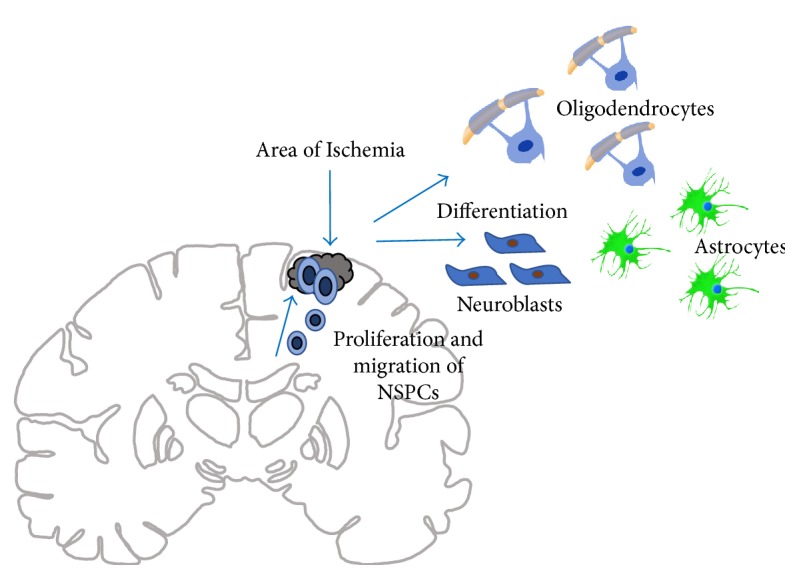
This figure demonstrates the process of neurogenesis following ischemic injury in a coronal section of the brain. Endogenous NSCs proliferate and migrate from the SVZ to areas of ischemic injury. Once they are outside the SVZ, they are able to undergo differentiation into oligodendrocyte progenitors, astrocytes, and neuroblasts.

Endogenous NSCs, aside from providing new neurons for olfactory and hippocampal neurogenesis under normal physiological conditions, also proliferate and migrate to areas after ischemic brain injury. Cerebral ischemia evokes a proliferation and migration response of NSCs towards the area of injury where they then differentiate into oligodendrocyte progenitors, astrocytes, and neuroblasts (Figure 2) [28].

It has been established that brain ischemia induces neurogenesis by activating neuronal migration through the injured area via secretion of neurotrophic factors such as brain-derived neurotrophic factor (BDNF), vascular endothelial growth factor (VEGF), cytokines like monocyte chemoattractant protein (MCP-1), and macrophage inflammatory protein (MIP-1). In addition, the natural inflammatory process in response to injury is able to induce NSC enrollment.

Neuroinflammation following ischemic stroke augments chemokine production by astrocytes and microglia [29, 30]. A recent study by Magnusson et al. suggests the reduction in NOTCH signaling pathways by astrocytes after recent ischemic stroke induces latent neurogenic programs. NOTCH1 is a gene which encodes a single transmembrane protein that plays a major role in cell fate. Attenuating NOTCH1 signaling to allow neurogenesis by striatal astrocytes may be useful for neuronal replacement following injury or cell death [31]. NSPCs were cultured from a gestational day 14 mouse embryo to study hypoxia-inducible factor-1*α* (HIF-1α). NSPCs were able to sustain a continuous level of HIF-1α, a crucial element of neural progenitor cells in responding to hypoxic events and affording neuroprotection from hypoxic events. In addition, HIF-1α was expressed in the SVZ and SGZ, suggesting adult neurogenic zones share similar characteristics of a developing embryonic brain [32].

Harms et al. showed that HIF-1α is necessary for NSC-induced neuroprotection in an oxygen-glucose deprivation culture model. HIF-1α gene deletion proceeds diminished VEGF expression along with Notch-*β*-catenin expression, negatively impacting endogenous NSC resistance to oxygen-glucose deprivation [33]. For the aspect of association between neurogenesis through angiogenesis or gliosis, an experimental study by Thored et al. exhibited how two hours of middle cerebral artery occlusion (MCAO) induced neural progenitor proliferation and neurogenesis in the SVZ, exhibiting permanency for four months after ischemic insult. Additionally, the route of neuroblast migration towards the damaged cortex demonstrated a higher vessel density than other areas (Figure 2) [34].

A study by Li et al. confirmed that endogenous stem cell response six weeks after cerebral ischemia in the SVZ involves production of oligodendrocyte progenitors and astrocytes. They also found that survivability of neuroblasts two weeks post-MCAO was less than oligodendrocytes and astrocytes (10% versus 15–20% and 59%, resp.) [28]. Another study by Kadam et al. using a rodent model in neonatal stroke also found similar results in the amount of SVZ-derived cells able to survive and become neurons [35]. These studies highlight the intricate steps to NSC proliferation, migration, and differentiation after ischemic injury.

## 5. Neurogenesis following Ischemia: Exogenous Stem Cells

In addition to using endogenous NSCs to induce neurogenesis in areas of ischemic injury, researchers can harvest, expand, and reimplant human stem cells in the area of damaged brain parenchyma as a form of cell replacement, regeneration, and repair. Ischemic insult causes damage to multiple different specialized cell types, and finding an option to repair and regenerate the entire neurovascular unit is the focus of ongoing studies. This section looks at the capabilities of exogenous stem cells in their ability to proliferate, migrate, survive, and differentiate. Large categories of exogenous stem cells include embryonic, neural, mesenchymal, and inducible pluripotent stem cells.

## 6. Embryonic Stem Cells

**Figure 3:**
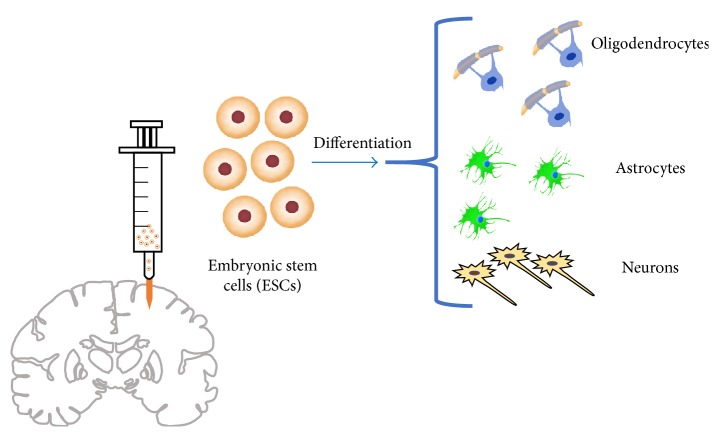
This figure demonstrates the effects of ESC transplantation into the brain. When ischemic stroke occurs, ESCs promote neuronal differentiation in both neurogenic and nonneurogenic regions, such as the striatum. ESCs are able to respond to environmental changes after ischemic insult and improve the capacity to promote neurogenesis.

Embryonic stem cells have been considered a source for promoting neuronal replacement because of their ability to respond to both extrinsic and intrinsic signaling towards specific neuronal differentiation [36]. Though it is somewhat unclear as to whether differentiation into neurons depends on the transplantation site, brain damage, or a default mechanism, researchers have studied which regions of the adult brain support neuronal differentiation of embryoid body cells derived from embryonic stem cells. Maya-Espinosa et al. compared neurogenesis in adult rat brain and postnatal day 24 rat. Neural uncommitted embryoid body cells differentiated into glia, neural precursors, and neurons in the adult rat brain in both neurogenic and nonneurogenic regions. They found that in neurogenic areas of the adult rat brain, including in the vicinity of the RMS and the cortex, neuronal differentiation, as opposed to astrocytes, was the preferred fate of the embryoid body cells. These results were helpful in determining that neurogenic conditions were not exclusively associated with juvenile brains. However, in contrast with the striatum of a young brain, there was less cell expression of neural biomarkers in the adult striatum. They found that after MCAO, regions that were not appropriate for neural differentiation were now able to promote differentiation, as evidenced by embryoid body cells giving rise to both astrocytes and neurons (Figure 3). These findings suggest that ischemic events promote neuronal differentiation of embryoid body cells in addition to attracting endogenous neural precursor cells to the injured area in adult rat brain [7].

Understanding the role of human embryonic-derived NSPCs has been investigated for improving stroke outcomes. A study by Rosenblum et al. sought to pretreat NSCs with BDNF, a growth factor that, as stated earlier, promotes nerve cell survival and function, and compare functional recovery with and without pretreatment of BDNF. They found that the BDNF-treated NSC group showed increased sensorimotor and neural recovery compared to the untreated and control groups over a one-month period following transplantation. They also found that the hippocampal region had a higher percentage of neuronal differentiation signaling, as well as increased overall neuroprotection due to the secretion of both VEGF, which promotes angiogenesis, and BDNF [37]. A study by Liu et al. found that after ischemic injury, transplantation of human embryonic NSCs into the lateral ventricle showed differentiation into neurons in the peri-infarct parenchyma and into oligodendrocytes and astrocytes in the corpus callosum. The human embryonic NSCs decreased ischemia-induced infarction after MCAO in rats and improved neural function [38]. In light of the benefits provided by embryonic stem cells in improving stroke outcomes and inducing neuronal differentiation, the general use of embryos for clinical stem cell transplantation poses more ethical obstacles than does the use of adult NSCs.

## 7. Exogenous Neural Stem Cells

Huang et al. experimented with NSCs in order to determine whether NSCs migrate into ischemic regions following stroke. Using a murine model, stroke was induced via MCAO. Subsequently, NSCs were injected into the hippocampus one day after stroke onset. The results indicated that only after one day of treatment, the cells migrated into the site of injury and the infarct volume was reduced. Treated mice fared better than their control counterparts in behavioral tests. The researchers hypothesized that an anti-inflammatory pathway led to this result. This study is one of the first demonstrating the short-term benefits of NSCs on behavior [39].

**Figure 4:**
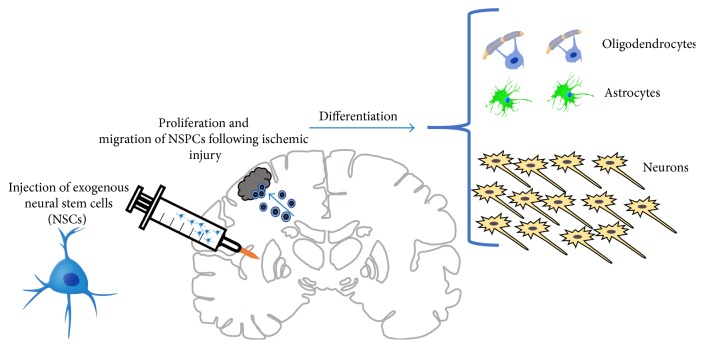
This figure demonstrates the effects of NSC transplantation following ischemic injury in a rat brain. Exogenous NSCs promote increased migration and proliferation of endogenous NSCs. In addition, there is increased differentiation into neurons compared to glial cells.

Using NSCs extracted from the hippocampus of fetal rats at 14 days gestation, scientists were able to isolate and culture the NCSs and, after inducing ischemic injury using a cerebral ischemia and reperfusion rat model, stereotaxically inject them into the left striatum. Immunofluorescent labeling showed proliferation of endogenous NSCs beginning day 3 poststroke. When comparing the amount of neurons to glial cells, the NSC transplantation group had less glial cell differentiation and more positive labeled cells for neurons compared to the control group with phosphate-buffered saline (Figure 4). Additionally, this study found functional improvements including less hemiplegia, smaller infarct volume, decreased nerve cell damage, and less apoptotic-positive cells [5].

A study by Cheng et al. found significant improvement in neurological deficits in MCAO rats compared to control using neonatal derived NSCs from mouse cerebellum and transplanting them via an intravenous grafting procedure to avoid surgical trauma. This study did not find a reduction in infarct size or volume, but they found migration abilities to the damaged area as well as increased proliferation of endogenous cells compared to control. They also noted that by day 28, though some NSCs had proteins specific for astrocytes or neurons, most had not yet differentiated [40]. These studies show promising capabilities for exogenously derived NSCs on improving the detrimental effects of ischemic stroke, including their important role in recruiting and activating more endogenous NSCs to assist with regeneration and repair.

## 8. Mesenchymal Stem Cells

**Figure 5:**
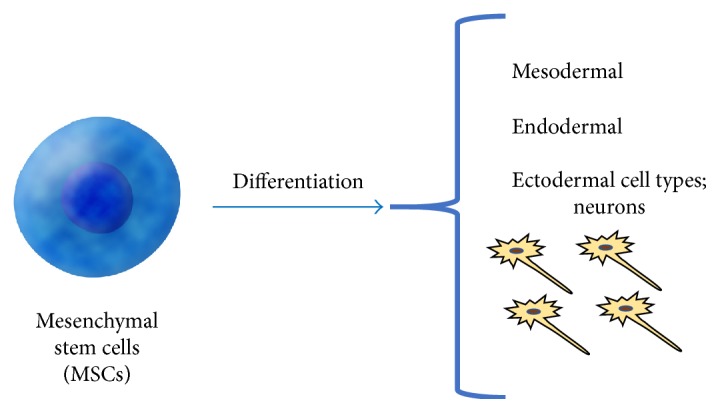
This figure demonstrates the differentiation capacities of mesenchymal stem cells. They are multipotent cells and can differentiate into mesodermal, endodermal, and ectodermal cell types. This includes the ability to become neurons, an important characteristic for the study of ischemic stroke and stem cells.

Mesenchymal stem cells (MSCs) are multipotent cells, with the unique capacity to differentiate into mesodermal, endodermal, and ectodermal cell types, including neurons. These cells are typically derived from mesenchymal tissues, including bone marrow and adipose tissue. This heterogeneous mixture of cells contributes to their ability to differentiate and proliferate (Figure 5) [41]. They are able to cross the blood-brain barrier and preferentially travel to damaged areas and reduce apoptosis, increase basic fibroblast growth factor, and promote endogenous cellular proliferation [42]. One study developed differentiated neuronal MSCs using the NOTCH intracellular domain and transplanted the committed cells into an ischemic area of adult gerbils, comparing their effects to noncommitted MSCs. Significant improvements were seen in recovery using neuronal MSCs; however, no synaptic connection occurred in endogenous cerebral cells [43].

A recent study by Liu et al. sought to improve the migratory capacity of bone marrow-derived MSCs after ischemic injury. It is well known that chemokines orchestrate cellular migration and SDF-1 contributes to recruitment of stem cells in ischemic areas of the brain with its receptor CXC chemokine receptor 4 (CXCR4) to aid in migration of bone marrow stem cells towards the injured area [44]. However, the majority of MSCs have intracellular CXCR4, and few express this receptor on the cell surface [45]. Though CXCR4 is highly expressed in the bone marrow, culture-expanded MSC's lose CXCR4 expression and responsiveness to chemokines, leading to decreased migration [46]. It is with this understanding in mind that Lin et al. performed preconditioning with tetramethylpyrazine (TMP), a pharmacologically active component extracted from a Chinese herb used for treatment of cerebrovascular and cardiovascular disease. It is known not only for its neuroprotective effect but also for its ability to regulate cellular migration, including neural precursor cells [47]. Preconditioning with TMP improved not only bone marrow-derived MSC migration towards ischemic areas but also increased CXCR4 mRNA and protein expression in vitro resulting in increased SDF-1 expression. Improved behavioral performance and angiogenesis in the region of the cortex undergoing ischemic insult was observed. The use of pharmacological agents may provide a more feasible way to improve the use and effectiveness of MSCs in the clinical setting in the treatment and recovery of neurological function after ischemic stroke [48].

Park et al. aimed to investigate the effects of multiple doses of MSCs compared to a single dose of stem cells following stroke. MSCs derived from human umbilical cord blood were transplanted following MCAO in a rodent model. 5 *μ*l of MSCs was administered on the second day after focal cerebral ischemia. A second treatment group received a total of 5 μl in separate dosages on the second and ninth days post cerebral ischemia. Functional outcomes were assessed and although motor dysfunction was found in both MSC groups, there was a decrease in infarct volume and an increase in neurons within the penumbral region. The repeated treatment did not show clear and significant advantages over the single treatment, suggesting that treatment is most effective when administered within the therapeutic window following stroke [49].

MSCs have been shown to play an important therapeutic role in modulating the immune response during transplantation. MSCs seem to inhibit antigen-specific T-cells and promote regulatory T-cells. Aggarwal et al. analyzed the immunomodulatory functions of human MSCs on different types of immune cells and showed that MSC induction in vitro resulted in a reduction in the proinflammatory cytokines TNF-*α* and IFN-*γ*, with increased production of the suppressive cytokine IL-10. Suppressive effects of MSCs were elicited through inhibitors of PGE_2_ synthesis which suggests that the increased PGE_2_ production from MSCs may play an important role in the mechanism of immunomodulation. An in vivo animal model is currently under investigation to better understand the complete mechanism of immunomodulation by MSCs [50]. The findings of this study show potential benefit in allogenic transplantation where recipients often develop graft-versus-host-disease due to reactive T-cells in the allograft [51].

Survival of MSCs is a significant hurdle to overcome for its intended use in regeneration of cells after ischemic insult. Retention rates of cells posttransplant in a porcine ischemic heart were no more than 6% after 10 days [52]. MSC survival in an immunodeficient rodent heart model was less than 0.4% after only four days [53]. The mechanism of poor survival is multifactorial but can be concisely explained by the harsh microenvironment in which the area of ischemia creates. MSCs struggle with inflammation, hypoxia, and oxidative stress due to inadequate nutrients and oxygen in the region [54]. Strategies aimed at improving survival include developing better ways to deliver MSCs to the ischemic area, preconditioning cells to better tolerate the microenvironment, and modifying the cells by genetic means [52]. Further investigation is needed to analyze and justify the most suitable improvements so that MSC therapy can remain a viable therapeutic option following ischemic stroke.

## 9. Inducible Pluripotent Stem Cells

Human inducible pluripotent stem cells (iPSCs) encourage potential restorative capabilities after ischemic stroke through their neuroprotective and neuroregenerative properties. However, application technique, adaptation, and optimization of iPSCs including their ability to differentiate may affect outcomes [55]. They are primarily generated from dermal fibroblasts, keratinocytes, lymphocytes, and hematopoietic stem cells by induced expression of transcription factors. Using gene expression and neuronal biomarkers, iPSCs were reported to generate cortical neural precursors in vitro [56]. An in vivo study was performed to assess whether iPSC-derived cortical neuronal progenitors, generated in vitro, survive transplantation and adequately differentiate in an injured adult brain. Human skin-derived iPSCs were found to have a neuronal phenotype differentiation capability in the somatosensory cortex and partially restored injured areas in an adult rat ischemic stroke model. At 2 months after transplantation the iPSCs not only proliferated but also survived, generating neurons with the same cortical phenotype observed in vitro. They expressed a cortex-specific biomarker TBR_1_ and exhibited layered patterning and projections, suggesting successful integration into the host brain. Electrophysiological data showed that the grafted cells acquired electrical properties, with the ability to fire action potentials [57]. Another study confirmed how grafted iPSCs can collect functional synaptic connections over a 6-month timeframe [58].

**Figure 6:**
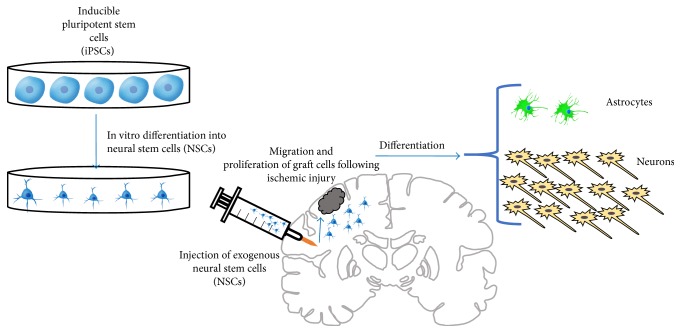
This figure demonstrates the capabilities of inducible pluripotent stem cells (iPSCs). Human iPSCs can be directed in vitro to the NSC phenotype. They are then injected into the cerebrum following MCAO and ischemic injury in rats. iPSCs are able to proliferate and differentiate primarily into neurons.

Jensen et al. aimed to test the survivability of NSCs derived from human iPSCs for treatment in an ischemic stroke model. The human iPSCs, derived from postnatal skin fibroblasts, were directed in vitro to the NSC phenotype and then were injected intracerebrally approximately one week following ischemia in adult rats. The amount of surviving graft cells was nearly double the number transplanted and expressed several neuronal biomarkers and neurite-like processes. Grafted cells integrated well and differentiated primarily into neurons in all members of the treatment group and rare astrocytes in half of the treatment group (Figure 6). No tumorgenesis was noted, and continued proliferation occurred one month after transplantation. Though this might suggest that the cell line was immature, the cells demonstrated remarkable survivability [59]. As a follow-up study regarding the optimal differentiation status of the stem cells, Jensen et al. in 2016 hypothesized whether grafting iPSCs depends on neural differentiation maturity status before treatment. The iPSC line was derived from postnatal human skin fibroblasts and differentiated to neural linages. 8-week old rats received intracerebral cell grafts at four different time points. The authors reported no significant difference among iPSCs at days 7, 28, 42, and 56 on the infarct size, behavioral recovery, microgliosis, astrocytosis, or neurologic outcome [60].

Neurotrophic factor-induced neuroregeneration has been associated with iPSCs. Chung et al. demonstrated how differentiation capabilities of mouse iPSCs also rely upon hypoxia-induced BDNF expression [61]. Cerebral transplantation of iPSCs showed beneficial outcomes in experimental stroke models, as evidenced by neuroinflammatory alterations, neuroplasticity enhancement, and neuronal replacement [62]. A recent study transplanted human iPSC-derived neuroepithelial-like stem cells into the area of ischemic injury in adult male rats and demonstrated the differentiation capacity through functional neurons as well as iPSC-derived cortical neurons. Ultrastructural signs indicated functional activity of synapses, including abundant synaptic vesicles and a wide range of synaptic contacts between grafted and host neurons. The majority of in vivo electrophysiological recordings at 5 months poststroke was made from grafted neurons and exhibited the same properties of mature neurons. The transplanted stem cells were able to gain afferent synaptic inputs from both the injured and uninjured cerebral areas and effectively maintained function from 6 weeks to 6 months following iPSC-derived stem cell transplantation [63].

Chen et al. examined the effects of iPSCs, generated from 13.5-day-old mouse embryonic fibroblasts, when transplanted in the subdural region of rodents with the help of fibrin glue. 8-week old adult rats underwent MCAO to induce ischemia. Direct injection of iPSCs into the brains showed 100% incidence of teratoma formation 4 weeks after transplantation. However, in the iPSC-fibrin glue group, no tumor formation or survival was observed 6 weeks after subdural transplantation in the ipsilateral cerebral hemisphere with MCAO. The results in the iPSC-fibrin glue group also included a smaller infarct size and improved motor function. In-depth analysis of cytokine expression demonstrated a decrease in proinflammatory cytokines coupled with an increase in anti-inflammatory cytokines, providing evidence for the efficacy of the iPSCs in the treatment of stroke using fibrin glue [64].

## 10. Combination/Cotransplantation Therapy

There has been a keen interest in using combination and cotransplantation therapies in treatment of ischemic stroke. A growing number of studies have shown encouraging results when combining single therapies to treat the aftermath of this often-debilitating phenomenon. As previously mentioned, it has been established that NSCs hold great potential in replacing cells that were lost due to ischemic stroke; however, the central nervous system (CNS) does not naturally provide an optimal microenvironment for transplanted NSCs to properly establish their intended purpose. To solve this issue of exogenous NSC survival, cotransplantation studies have been performed to evaluate whether there is an improvement in grafting efficacy of exogenous NSCs when other cells, such as astrocytes and/or microvascular endothelial cells, are cotransplanted.

**Figure 7:**
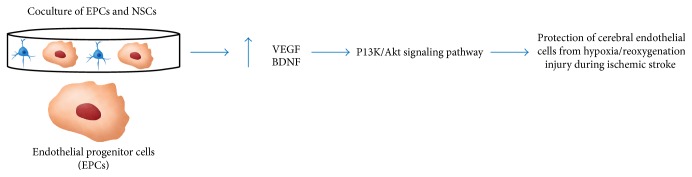
This figure demonstrates the effects of using cotransplantation as a method for stem cell therapy. An example of this approach is using endothelial progenitor cells (EPCs) and NSCs. Together, they create a synergistic effect that results in an increase in VEGF and BDNF. This activates the PI3K/Akt pathway that is thought to protect cerebral endothelial cells from hypoxia/reoxygenation injury during ischemic stroke.

Luo et al. used an animal model to explore whether astrocytes act to make the CNS microenvironment more suitable for differentiation of exogenous NSCs in an ischemic brain. Their results were encouraging, showing that animals who received cotransplantation of astrocytes exhibited a higher likelihood of exogenous NSC survival [6]. Another important study was conducted by Wang et al. who revealed an understanding of a potential mechanism behind the synergistic effect of endothelial progenitor cells (EPCs) and NSCs protecting cerebral endothelial cells (CECs) from hypoxia/reoxygenation-induced injury associated with ischemic stroke. This in vitro study demonstrated that coculturing EPCs with NPCs resulted in an increase in VEGF and BDNF, which further activated the phosphatidylinositol-3-kinase (PI3K)/Akt pathway that is thought to protect CECs during ischemic stroke (Figure 7) [65].

To explore the potential benefits of cotransplantation further, an animal model study was conducted by Cai et al. who revealed that cotransplanting astrocytes and brain microvascular endothelial cells (BMECs) together with hippocampal NSCs improved memory deficits in ischemic stroke animal models. Furthermore, this improvement in memory was greater in animals that received both astrocytes and BMCEs compared to those that received one or the other along with the exogenous NSCs [66].

Using a tissue engineering approach, Zhang et al. demonstrated the beneficial effects of a combined treatment of plasma scaffold with MSCs. A rodent model of ischemic stroke was performed via MCAO. Three weeks later, the treatment was administered. The combined treatment group, consisting of the scaffold and MSCs, and the single treatment group of only MSCs showed better signs of recovery than the vehicle group. However, it is notable that the combined scaffold-MSCs showed more positive results than the single treatment group. This unique approach led to an improvement of motor function and a reduction in the amount of atrophy, further supporting the therapeutic benefits of MSCs [67]. A 2017 study by Augestad et al. studied the effects of cografting NSCs with olfactory ensheathing cells. These cells are a special type of glial cell with neuroprotective and angiogenic capabilities that may assist in graft survival. Using an MCAO rat model, they found extensive vascular remodeling and even more NSC movement towards the infarct border [68].

Stem cell clinical trials are well under way and showing beneficial outcomes. Qiao et al. assessed safety and feasibility of cotransplanting NSCs and MSCs into the brains of patients that experienced ischemic stroke. Although only eight patients were enrolled in this study, the results were encouraging as the patients exhibited an improvement in neurological function and disability levels. Furthermore, none of the patients experienced tumorigenesis when reevaluated during their two-year follow-up appointments [69]. The results of this study are encouraging; however, larger samples, extensive follow-up, and standardized study design methods, such as utilization of control groups, are required to further explore these observations (Table 1).

## 11. Stem Cell Tracking Using Magnetic Resonance Imaging

It is of specific importance to be able to track and monitor the dynamics of endogenous stem cells in ischemic stroke. Typically, these NSCs are located in the SVZ of the lateral ventricle and the SGZ in the dentate gyrus of the hippocampus [70]. After ischemic stroke, NSCs are triggered to proliferate and migrate towards the injured region of the cortex, and it is this process that in vivo tracking aims to visualize, as well as provide insights into the SVZ under ischemic conditions [71]. In order to effectively use stem cells as a reliable tool in the clinical setting, there needs to be a method of tracking and long-term monitoring of cell acceptance, growth, distribution, differentiation, and cell survival of the transplanted stem cells [8].

Intracellular magnetic labels such as superparamagnetic iron oxide nanoparticles (SPIONs) have their surface modified to facilitate cellular uptake, and they work well for tracking experiments because of their molar relaxivity and can induce internalization of the contrast medium without interrupting cellular functions [72]. Zhong et al. found that in vivo targeted magnetic resonance imaging (MRI) of endogenous NSCs in a normal adult rodent brain could be achieved using anti-CD15 antibody-conjugated superparamagnetic iron oxide nanoparticles (SPIONS) as the molecular probe [73]. This method is able to overcome the shortcomings of using a nonspecific SPION or a ferritin-based reporter gene, including low imaging sensitivity, nontargeting, and toxicity [74].

Zhang et al. explored the use of anti-CD15 mAB SPIONS, which previously showed benefits in being able to monitor endogenous NSC migration, as the imaging probe in targeted tracking of activated endogenous NSCs expressing the CD15 antigen on the surface of NSCs after cerebral ischemia. Their findings included proliferation of endogenous NSCs without migration towards the infarcted lesion, possibly due to an 8-day follow-up post ischemic stroke, or a lower imaging sensitivity of SPIONS compared to micron-sized particles of iron oxide (MPIOs). In addition, by using the CD15-positive subpopulation of NSCs, not as many NSCs were visualized, as there are far less of this subtype than the nonspecific MPIO-labeled cells, which includes NSCs as well as neuroblasts, astrocytes, progenitor cells, and mature neurons [71]. MPIOs are found in microglia, ependymal cells, and oligodendrocyte progenitor cells in addition to being in NSCs, thereby providing a nonspecific tracking method [75]. Despite several limitations of anti-CD15 mAB SPIONS for tracking stem cells, this is a more recent approach to effectively track and monitor endogenous stem cells in vivo.

iPSC-derived neural precursors offer an exogenous source for stem cell transplantation therapy in CNS disorders. A study investigated the effect of two different contrast agents on neural precursor cell proliferation and differentiation capability using silica-coated cobalt zinc ferrite nanoparticles and SPIONs coated with poly-l-lysine (PLL). They found that PLL-coated SPIONs did not have any significant negative effects on cell proliferation or differentiation in any dose, as opposed to the silica-coated cobalt zinc ferrite nanoparticles that negatively impacted cell proliferation. PLL-coated SPIONs were found effective for noninvasive cell tracking and show promising use in future neural cell therapy-based in vivo applications for different disease models [76].

A study in 2013 designed a mesoporous silica-coated SPION to utilize for neural progenitor cell MRI. Compared to fluorescent dense silica-coated SPIONs, a commercially available contrast agent, the mesoporous silica-coated SPIONs had improved uptake efficiency potentially due to their less negative surface charge. It also had improved cell internalization over SHU555A, another commercially available contrast agent used for cell imaging [77]. After an incubation period of 3 hours, no direct cytotoxic effects could be found in the short term, but viability did decrease after 24 hours of incubation, with similar cytotoxicity levels as noted in SHU555A, likely due to high intracellular iron concentrations [78]. Using the noncytotoxic conditions of 10 micrograms Fe/ml for 2 hours, cell proliferation was not impacted. Researchers then used these criteria to perform intracerebral and intravenous injections of labeled progenitor cells in MCAO mouse model. Both methods were able to show that the transplanted progenitor cells migrated to the ischemic site, with cell clusters detected near the lesion boundary [79] (Table 2). Advances in stem cell tracking using MRI following ischemic injury are well underway, and new molecular probes are being testing in vitro with hopes of providing additional tracking options. Currently, after searching on clinicaltrials.gov using search terms “stroke and stem cell tracking,” no clinical trials on tracking methods are being performed at this time. Studies that elucidate consistent results regarding effectiveness and safety in animal models will continue to lay the groundwork for future clinical trials.

## 12. Conclusion

Basic science research on stem cell treatment of stroke is a necessity and may change the lives of millions around the world burdened by the effects of ischemic stroke. The progress done so far in animal research has led to multiple clinical trials showing the safety and benefits of stem cells with recovery from ischemic stroke. Clinical trials are underway, most within phase I or phase II and focusing on MSCs or cotransplantation methods. Basic science studies continue to publish results on the benefits of transplanting stem cells after stroke to improve recovery, infarct size, and reduce apoptotic events and neurodegeneration. Various new methods are being tried, including cotransplantation and preconditioning with pharmacologic agents known to induce angiogenesis and improve receptor binding for neuronal migration and inducing more endogenous stem cells to migrate. Finding consistent methods to promote not only neuronal differentiation but also adequate migration to the area of infarct is a critical issue in the field of stem cell transplantation. The challenges in the area of improving tracking methods include length of tracking using current methods and the risk of provoking an immune response or affecting intrinsic properties of stem cells. Monitoring the process of cell acceptance, growth, migration, differentiation, and cell survival of injected cells is important to be able to analyze and understand the process after stem cell transplantation. Noninvasive strategies to study neurogenic mechanisms of stem cells will benefit the development of future studies and therapies in the field in search of improving the overall utilization of NPSCs in the treatment and recovery of ischemic stroke.

## Figures and Tables

**Table 1:** Current completed phase I and II clinical trials on stem cell and ischemic stroke.

Stem cell completed clinical trials for ischemic stroke					
Authors	NCT	Stage of trial	Type of stem cells used/mode of delivery	Primary outcomes	Results
Kalladka et al. 2016 [9]	01151124	Phase I	NSCs: CTX-DP drug product/stereotactic ipsilateral putamen injection	Incidence of adverse events	No adverse events were seen NIHSS improvement ranged from 0–5 (secondary outcome)
Qiao et al. 2014 [69]	NA	Phase I	Cotransplantation with neural stem/progenitor cells and mesenchymal stromal cells/IV	Safety and feasibility	No evidence of neurological deterioration or neurological infection
Prasad et al. 2014	02425670	Phase II	BM mononuclear cells/IV	Functional ability-modifiedBarthel Index score	No significant difference between BMSC versus control in Barthel Index score
Banerjee et al. 2014	00535197	Phase I	CD34^+^ stem cells/intra-arterial	Safety	Safe and feasible
Savitz and Sean 2014	00859024	Phase I	Autologous mononuclear bone marrow cells/IV	Adverse events	No study-related adverse events

IV: intravenous; BMSC: bone marrow stem cells; NIHSS: National Institute of Health Stroke Scale; NCT: National Clinical Trial Number.

**Table 2:** The 3 most common studies that tested various molecular probes to track stem cells in both normal and ischemic brain.

In vivo tracking using magnetic resonance imaging						
Author	Target	Probe delivery method	Molecular probe	Location	Signal time	Findings
Zhang et al. 2016 [71]	Endogenous NSCs in vivo ischemic stroke adult rats	Stereotactic injection	Anti-CD15 antibody SPIONS	Intraventricular SVZ and RMS regions in adult mouse brain following MCAO	Detected day 1–day 8	(i) Proliferation of endogenous NSCs but no migration towards infarction lesion (ii) Findings may be due to short-term follow-up of only 8 days (iii) SPIONs have less imaging sensitivity than MPIOs (iv) Migration to OB along RMS observed after ischemic stroke (v) Introduction of heterologous antibody risk host immunological response (vi) Many other phenotypes undetected

Zhong et al. 2015 [73]	Endogenous NSCs in vivo normal adult rats	Stereotactic injection	Anti-CD15 antibody SPIONs	Intraventricular SVZ and RMS regions in adult mouse brain	Detected 1 day after injection, lasted 7 days total	(i) Small size, low artifact (ii) Increased specificity for NSCs (iii) Can track highly active areas such as OB surface binding less likely to affect biological behavior of cells (iv) Introduction of heterologous antibody risks host immunological response (v) Many other phenotypes undetected

Zhang et al. 2013 [79]	Exogenous neural progenitor cells in vivo ischemic stroke adult mice	Intravenous and implantation into hemisphere contralateral to stroke	Fluorescent mesoporous silica-coated superparamagnetic iron oxide nanoparticles	Right hemisphere following MCAO	Detected and analyzed 1–3 days after injection	(i) Cells injected both intracerebrally and intravenously could be seen migrating to ischemic sites of MCAO mice (ii) Migrated cells/cell clusters were detected nearby the lesion boundary (iii) Migrated cells reduced the MR signal intensity in ischemic region 3 days after injection
